# Mammalian Cell Interaction with Periodic Surface Nanostructures

**DOI:** 10.3390/ijms23094676

**Published:** 2022-04-23

**Authors:** Petr Slepička, Silvie Rimpelová, Vladimíra Svobodová Pavlíčková, Nikola Slepičková Kasálková, Klaudia Hurtuková, Dominik Fajstavr, Václav Švorčík

**Affiliations:** 1 Department of Solid State Engineering, University of Chemistry and Technology Prague, 166 28 Prague, Czech Republic; nikola.kasalkova@vscht.cz (N.S.K.); klaudia.hurtukova@vscht.cz (K.H.); dominik.fajstavr@vscht.cz (D.F.); vaclav.svorcik@vscht.cz (V.Š.); 2 Department of Biochemistry and Microbiology, University of Chemistry and Technology Prague, 166 28 Prague, Czech Republic; vladimira.svobodova.pavlickova@vscht.cz

**Keywords:** polymer, excimer laser, periodic pattern, elemental analysis, LIPSS, human cells, cytocompatibility, cell guidance

## Abstract

Here, we report on the nanopatterning of different aromatic polymer substrates achieved by KrF excimer laser treatment. The conditions for the construction of the laser-induced periodic surface structures, the so-called LIPSS pattern, were established by optimized laser fluence and a number of pulses. The polymer substrates were polyethylene naphthalate (PEN), polyethersulfone (PES), and polystyrene (PS), which were chosen since they are thermally, chemically, and mechanically resistant polymers with high absorption coefficients at the excimer laser wavelength. The surface morphology of the treated substrates was investigated by atomic force microscopy and scanning electron microscopy, and the roughness and effective surface area on the modified samples were determined. Elemental concentration was characterized by energy-dispersive (EDX) analysis, surface chemistry was determined with X-ray photoelectron spectroscopy (XPS). The samples with the formation of LIPSS induced by 10 mJ·cm^−2^ with 1000, 3000, and 6000 pulses were used for subsequent in vitro cytocompatibility tests using human cells from osteosarcoma (U-2 OS). The LIPSS pattern and its ability of significant cell guidance were confirmed for some of the studied samples. Cell morphology, adhesion, and proliferation were evaluated. The results strongly contribute to the development of novel applications using nanopatterned polymers, e.g., in tissue engineering, cell analysis or in combination with metallization for sensor construction.

## 1. Introduction

Surface micropatterning may be used for the spatial arrangement of single cells by fabrication of cell-adhesive spots surrounded by cell-repellent surfaces. This contact-based single-cell trapping is an easy and cheap method for high-throughput studies. Commonly used biomimetic materials and cell adhesion molecules for adhesive regions are fibronectin [1], laminin, collagen [2], vitronectin, and poly(L-lysine) [3]. As hydrophilic polymers for cell-repellent surface modification, polyethylene glycol (PEG) [4,5], polyvinyl alcohol (PVA) [6], or alkanethiol [7] may be applied. Different strategies including microcontact printing [8], ink-jet printing [9], and photopatterning [10] have been developed to produce chemical surface patterns. Interaction between single molecules and microscopic structures [11,12] is strongly affected by new properties and functionalities of the matter, also considering various physical or chemical phenomena, as well as chemical and biological processes, which very often occur at the nanoscale [13]. Therefore, even when biological and chemical systems seem to be in equilibrium on the macroscopic scale, it often exhibits a complex set of reactions at the nanoscale. Nanofabrication methods for controlling cell–substrate interactions and cell behavior play a crucial role in tissue engineering [14,15]. Surface plasmons were recently used as a strong tool to measure cell–substrate interactions [16]. Commonly used materials such as polymers are very often applied as substrates in medicine and biology. Moreover, the detailed knowledge of the surface morphology and chemistry may also serve for specific biological applications in the field of antimicrobial materials [17], for cell growth guidance [18], and for a deeper understanding of fundamental phenomena and processes which may occur between the living organisms (cells or bacteria) and activated or nanopatterned polymer surface [19].

Small changes in the local nanoscale environment often imply major changes also in the macroscopic properties or behavior of substances, macromolecules, cells, and organisms [20], which may be subsequently analyzed with microscopic techniques such as scanning electron microscopy [21] or atomic force microscopy [22]. The effect of such changes may be significantly amplified by the presence of laser-induced periodic surface structures, so-called LIPSS. This periodic nanostructure may enhance physico-chemical reactions with the surface or interactions with living cells. In addition, subsequent metallization of the surface with, e.g., noble metals, is also possible. This metallization may enhance the plasmonic properties of such structures as the most effective tool for direct visualization, sensitive detection, and modification of molecular interactions in vitro or in vivo in living organisms. In the past, plasmonic particles were used for detecting small numbers of molecules and ions [23,24], monitoring antibody−antigen interactions [25], and surface-enhanced Raman scattering (SERS) [26].

Nanostructures acting like nano heaters can be used to provide contrast in photothermal therapy [27]. The metalized surface may also induce a strong antibacterial effect [17], which opens a wide possibility to study interactions of specific bacterial strains and their responses to different metals or metal oxides, and more importantly to different surface morphology consisting of various patterns ranging from periodic lines [28], dots [29], to even, e.g., nanopatterned primary microstructures such as honeycomb-like microstructures [30]. The appearance of localized plasmon modes enables the focusing of light in nanoscale volumes, i.e., the localization of electromagnetic energy due to the formation of highly enhanced hot spots at the surface of the nanostructures. Plasmon modes can also couple to the electromagnetic fields emitted by molecules and atoms in the vicinity of the nanostructures. Therefore, there will be an effect whether the vicinity consists of biological material such as bacterial or mammalian cells or only of cell culture medium. The interaction distances are typically in the order of a few hundreds of nanometers. This should also enable the assessment of the adhesion strengths of a microorganism to the noble metal surface. Furthermore, plasmonic structures efficiently convert absorbed light into heat, resulting in a temperature increment localized in the nanosurroundings of the structures [7]. The heating may have positive or negative effects on the adsorption of biofilms to the nanowires. The nanopatterned surface plays an important role in cell guidance. Understanding cellular interactions with nanomaterials leads to a rational design of medical nanodevices [31]. Nanopatterned interfaces for the control of cell behavior [32], not only polymers but also special metals such as titanium, may be nanostructured and used for cell guidance [33]. Micro and nanofabrication methods to control cell–substrate interactions and cell behavior may be used for surface fabrication of various types of materials [34] with the ability to guide several cell types [35,36,37,38].

In this study, we focused on the mapping of LIPSS formation induced by KrF excimer laser treatment on aromatic substrates PEN, PS, and PES. We studied the impact of laser fluence and the number of laser beam pulses on material surface morphology, roughness, surface area, and chemistry using different analytical methods. The main aim of this article is a study of cell alignment, cell adhesion, and proliferation study, where different aromatic polymers have been used. Moreover, the study of the surface area of the patterned surface may play a crucial role in cell filopodia development and shape. We have aimed mainly at material properties in combination with appropriate input parameters for the creation of the surface of desired LIPSS properties either with particular elemental concentration or morphology construction in this article.

## 2. Results and Discussion

The main purpose of this study was to determine the ability of a LIPSS pattern on a particular polymer substrate to affect cell growth and to study the possibility of cell guidance. From our previous experience in the field of LIPSS preparation, we have focused on aromatic polymers with the ability to absorb the particular laser wavelength and, thus, to create periodic pattern structures on their surfaces, if specific conditions regarding the laser polarization, angle of laser beam incidence, and laser beam intensity are fulfilled [39].

### 2.1. Scanning Electron Microscopy

**Figure 1:**
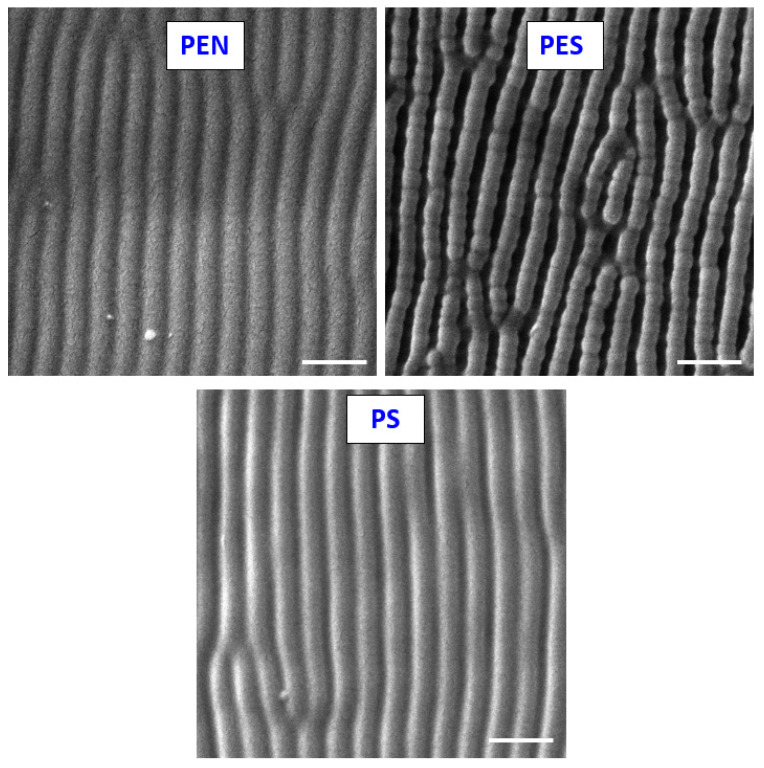
Scanning electron microscopy images of PEN, PES, and PS foil treated by KrF laser with the laser fluence of 10 mJ·cm^−2^ with 6000 pulses. The white line represents 500 nm.

The SEM technique was used to confirm and extend the knowledge about the LIPSS formation. As a complementary technique, we have used the EDX analysis, which was used either for elemental analysis of the modified polymer surface and/or elemental mapping, which can be further complemented with goniometry analysis. The surface morphology of the studied polymers with scanning electron microscopy confirmed the LIPSS pattern formation for all studied polymers (Figure 1). 

As is obvious from the studied samples, the sample’s periodicity was observed for a combination of 6000 pulses and 10 mJ·cm^−2^. The initial stages of LIPSS pattern formation (1000 pulses and 3000) are connected with a dramatic increase in surface roughness and the formation of periodic patterns with a significantly lower height of the pattern. The LIPSS formation was the most pronounced even for a short number of pulses on PES, on which even at 1000 laser beam pulses, the LIPSS was detected, but with significantly lower height, not exceeding 10 nm. PS was different from other studied polymer representatives; the application of only 3000 pulses led to the formation of LIPSS. The periodic surface pattern significantly affects the cell−polymer interaction, mostly in combination with wettability and elemental concentration changes, as will be discussed further in the text.

The difference in the primary chemical composition of all studied polymers plays a crucial role in further change in elemental concentration. We have focused on particular changes in the elemental concentration of patterns constructed under irradiation with the fluence of 10 mJ·cm^−2^ while changing the number of laser beam pulses. Since, from the previous experiments, it is known that the laser beam fluence has a significant effect on the surface of the treated polymer [40,41], in this study, we have focused on the particular effect of several laser beam pulses while the laser beam fluence remained constant. We have used EDX analysis, which gives us the information not from the very top of the polymer pattern, but up to several hundreds of nm. The data on elemental concentration changes were followed by a subsequent study on cell interaction with laser beam patterns.

**Figure 2:**
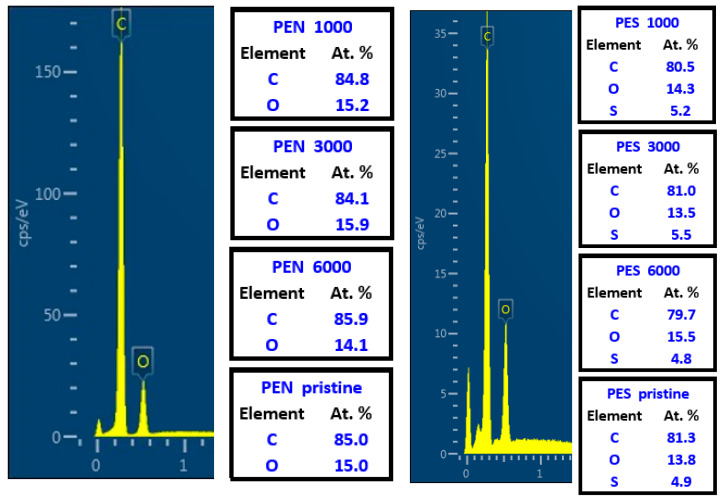
Spectra from energy-dispersive X-ray spectroscopy (EDX) analysis and elemental atomic concentrations (carbon, oxygen, sulfur) for PEN and PES samples treated by KrF laser with the laser fluence of 10 mJ·cm^−2^ and 1000, 3000, and 6000 pulses.

**Figure 3:**
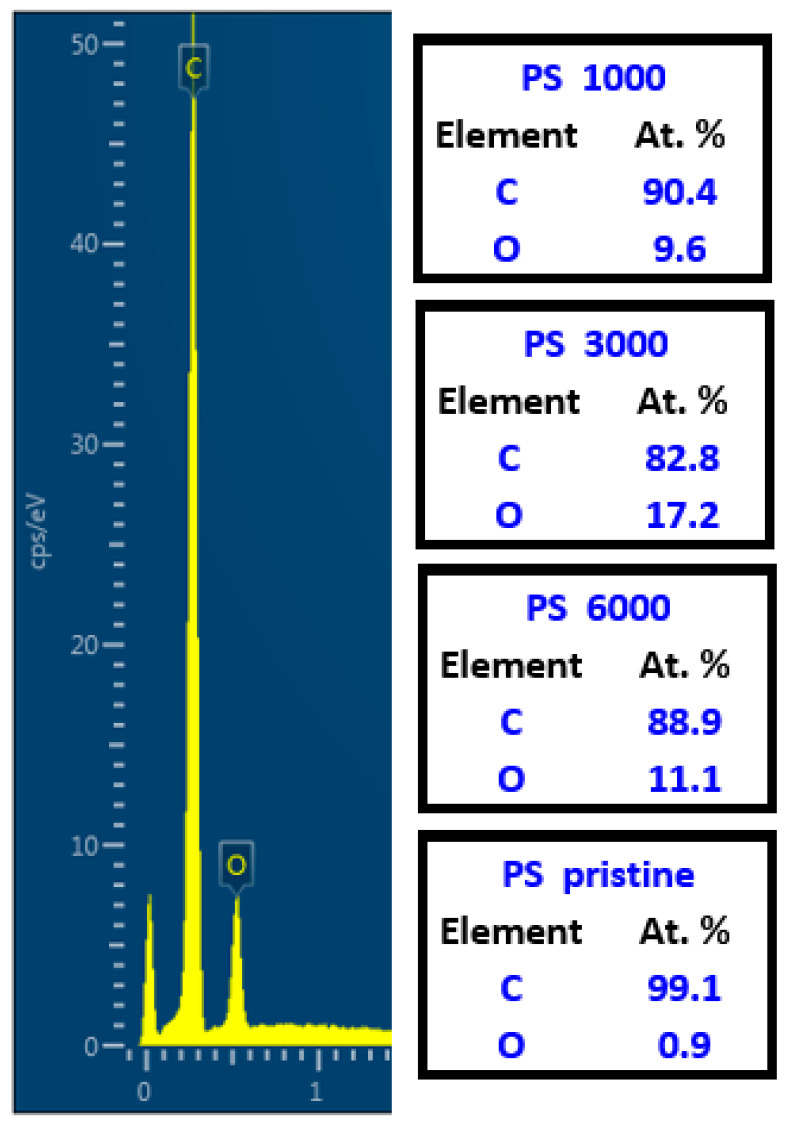
A spectrum from energy-dispersive X-ray spectroscopy (EDX) analysis and elemental atomic concentrations (carbon, oxygen) for PS samples treated by KrF laser with the laser fluence of 10 mJ·cm^−2^ and 1000, 3000, and 6000 pulses.

As is obvious from Figure 2, the laser beam exposure led to slight variations in surface oxygen concentration for PEN and PES irradiated with the excimer laser. As is obvious from the EDX spectra of PES, the largest increase in oxygen concentration was determined for the sample treated with the highest number of laser beam pulses (Figure 3). However, a significant increase in oxygen due to excimer laser treatment was detected for PS, for which the oxygen increased up to 17.2% at 3000 pulses. This increase is connected with the movement of polymeric chains during the laser exposure, where the polymer is close to the glass transition temperature. The surface of the chain is oxidized, and during the movement of polymeric chains at this stage of the process, the additional chains “from the bulk” are further oxidized until the pattern formation is finished. One has to point out that these results are different from those observed by XPS analysis, which we have acquired in our previously published studies [29,40,41], and also will be shown further. The difference arises from the depth of EDX detection, which is up to 100 nm. From the data that we have presented and compared with our previously measured spectra with the XPS technique, we conclude that the oxygenation takes place mostly on the very top of the treated polymers (up to 10 atomic layers). The EDX data, particularly fluctuations in oxygen concentrations, confirm that even the “bulk area” of the ripple pattern is not significantly affected by the oxygen increase. The highest oxygen increase was observed for PS, for which the same trend was determined previously by Nedela [42].

### 2.2. Atomic Force Microscopy

Primary parameters affecting the possibility to guide cell adhesion and growth are the width and height of a ripple pattern. As it was previously described for several aromatic polymers [40,41,42,43,44,45,46,47], these parameters are also partially influenced by the type of substrate, on which the excimer laser exposure is applied; this effect lies within the material parameter refractive index, usually referred also as modified refractive index [39]. 

Thus, even when the same set of input laser conditions are used for chemically different polymers (but still those with aromatic rings in the structure), the output dimensions of prepared laser-induced periodic surface structures (LIPSS) may differ, and the main parameter affected is the pattern width. Therefore, at the beginning of this study, we focused on precision in surface parameter determination, which was determined by atomic force microscopy.

**Figure 4:**
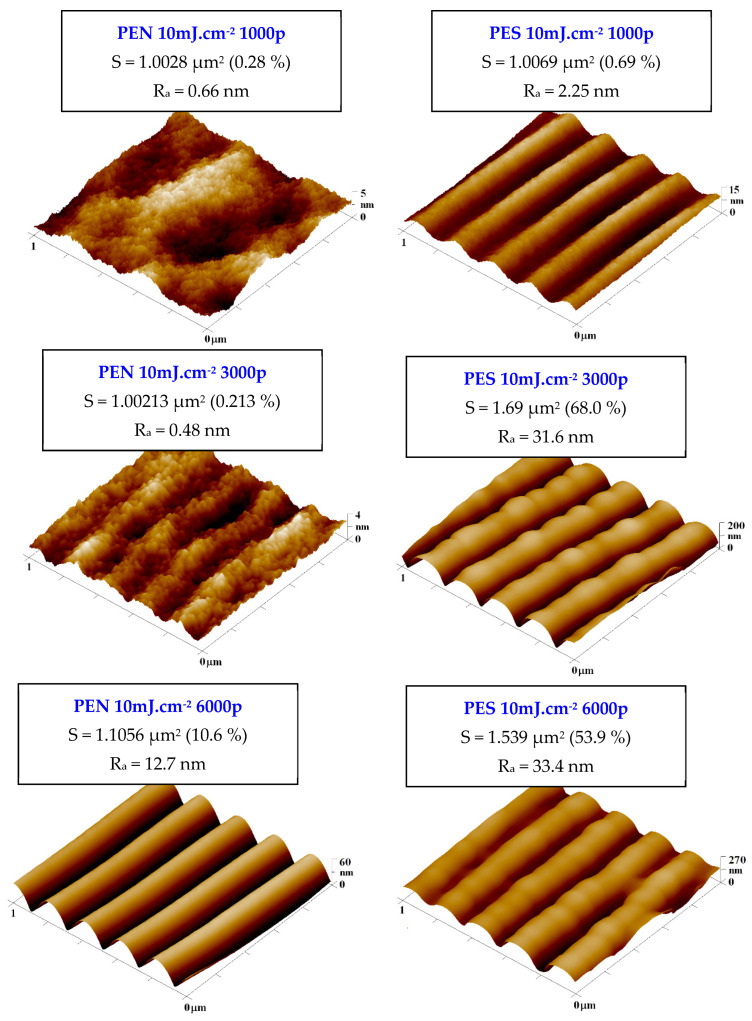
Atomic force microscopy images of PEN and PES foils treated by KrF laser with the laser fluence of 10 mJ·cm^−2^ and 1000, 3000, and 6000 pulses. The Ra represents average surface roughness and S the effective surface area.

As is obvious from Figure 4, the material surface morphology, LIPSS height, and width differed for a particular type of polymer; however, the dimensions of the pattern for the highest number of laser beam pulses were not significantly different among the applied polymeric substrates. We have chosen the region of 1 × 1 μm^2^ for AFM to have a detailed representation of the exposed area. With the particular morphology of the LIPSS pattern, we introduced also surface roughness (R_a_), effective surface area (S_a_), and most importantly, pattern height and pattern width. We have determined the following parameters for particular polymers, (pattern width)/(pattern height): PEN/(212.1 ± 2.0)/(44.4 ± 2.0); PES/(225.1 ± 9.5)/(107.6 ± 7.7); PS/(203.9 ± 3.4)/(52.4 ± 5.6).

**Figure 5:**
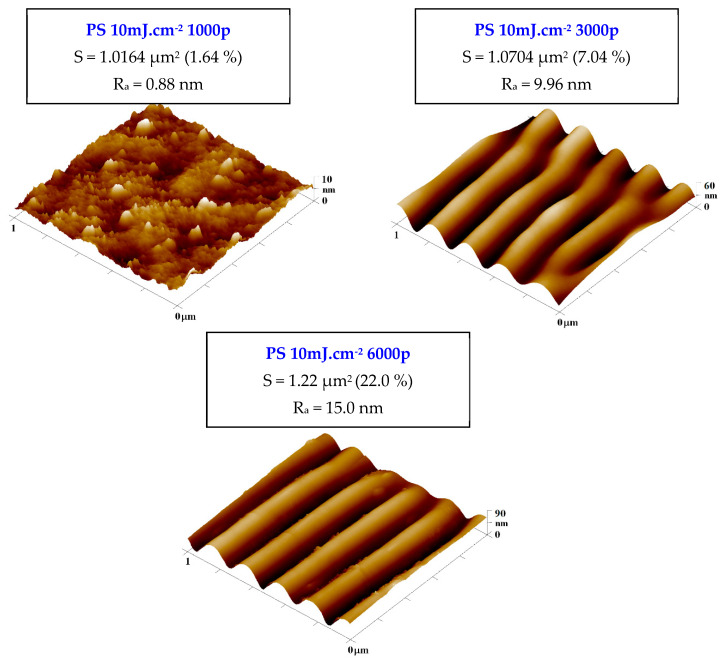
Atomic force microscopy images of PS foil treated by KrF laser with the laser fluence of 10 mJ·cm^−2^ and 1000, 3000, and 6000 pulses. The Ra represents surface roughness and S the effective surface area.

As is obvious from Figure 5, the pattern properties were significantly affected by the number of laser beam pulses and the integral laser beam dose. The optimal number of laser beam pulses can be considered as 6000, at which the LIPSS pattern is the most homogeneous with the lowest ratio of secondary periodicity [39,41]. The LIPSS pattern on all studied polymers had a width not lower than 230 nm, and the properties of the pattern slightly differed both in width and more efficiently in its height, especially for PES, for which also a strong significant secondary periodicity was observed. For all studied polymers, we have also observed the secondary periodicity (larger waves perpendicularly oriented to the primary pattern) [39]. However, for the cell guidance, this phenomenon should not have a negative effect—on the contrary, it should improve the cell attachment affecting the interaction of cell filopodia with the polymer surface. 

### 2.3. Wettability

**Figure 6:**
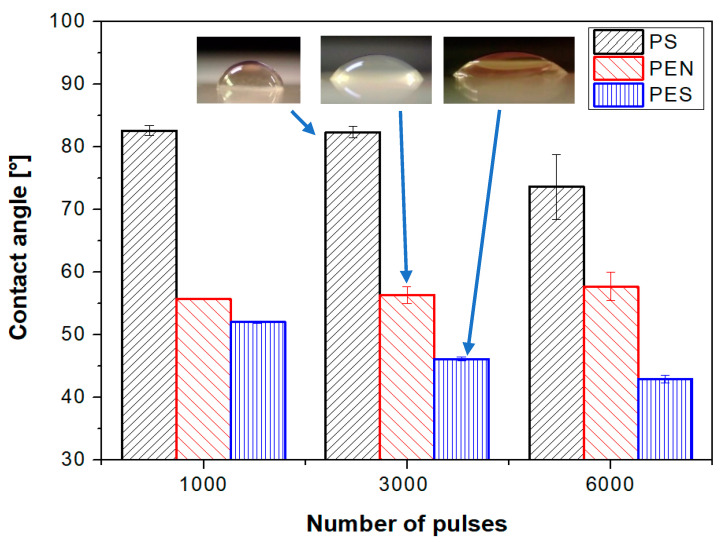
Contact angles of laser-treated polystyrene, polyethylene naphthalate, and polyethersulfone. The laser exposure of 10 mJ·cm^−2^ with 1000, 3000, and 6000 pulses was applied.

The contact angle determination was conducted for all studied polymers, for which the effect of the number of laser beam pulses on the surface wettability was detected. The values of contact angles for pristine polymers were detected to be as follows: pristine PEN (61.5°), PS (87°), and PES (74°). As is obvious from Figure 6, the excimer laser exposure led to significant changes in surface wettability, the changes were determined to be more pronounced with an increasing number of laser beam pulses. The largest decrease in a contact angle compared to pristine polymer was observed for polyethersulfone; for this polymer, the significant dependence of wettability on the number of laser beam pulses was also observed.

It can be also concluded that when a lower number of laser beam pulses is applied, the difference between pristine and laser-treated foil diminishes for all studied polymer foils. This phenomenon is connected with the pattern formation process, in which at the primary stages of pattern formation, the distribution of polymer mass due to surface diffusion occurs, but not significant chemistry or morphology changes. If the number of laser beam pulses increases, the oxygen-containing groups are present on the surface in concentrations affecting the wettability of the treated polymer, which is introduced both in Figure 4 and Figure 5. The surface physico-chemical changes may affect both the material cytocompatibility on the surface and more importantly the cell guidance. Both phenomena will be discussed in the following section.

### 2.4. XPS Analysis

**Figure 7:**
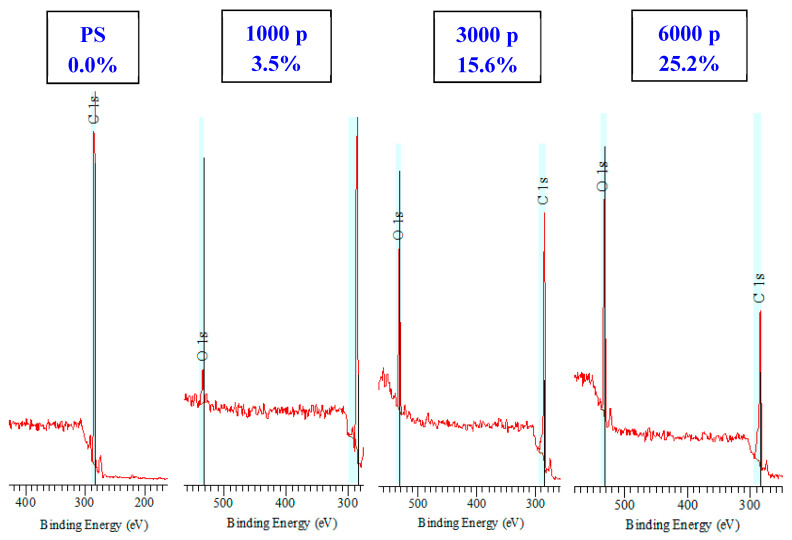
XPS spectra of pristine and laser-treated polystyrene. The laser exposure of 10 mJ·cm^−2^ with 1000, 3000, and 6000 pulses was applied. The oxygen atomic concentrations are introduced for particular samples.

We have determined the surface chemistry on the pristine and laser-treated samples for all applied numbers of laser beam pulses. We have focused on the changes in surface oxygen concentration, since they may affect the cytocompatibility and wettability. The XPS spectra of laser-treated and pristine polystyrene polymer are introduced in Figure 7. As is obvious from this set of spectra, the laser treatment induced a significant increase in oxygen concentration with an increase in the number of laser beam pulses. Pristine polystyrene does not have any oxygen in its chain, and none was detected on its surface either. The maximum atomic oxygen concentration of 25.2% was detected on polystyrene treated with 10 mJ.cm^−2^ and 6000 pulses. 

As is obvious from Table 1, a dramatic increase in surface oxygen was detected for polystyrene treated with an excimer laser. The increase in oxygen concentration was also observed for PEN polymer; the main increase was observed for 1000 pulses, and with a further increase in several laser beam pulses, only a mild increase in oxygen concentration on the PEN surface was observed. On the contrary, for PES polymer, even an increase in oxygen after exposure with 1000 pulses was observed, and a further increase led only to fluctuation in the surface oxygen concentration.

### 2.5. Cell−Material Interaction

**Figure 8:**
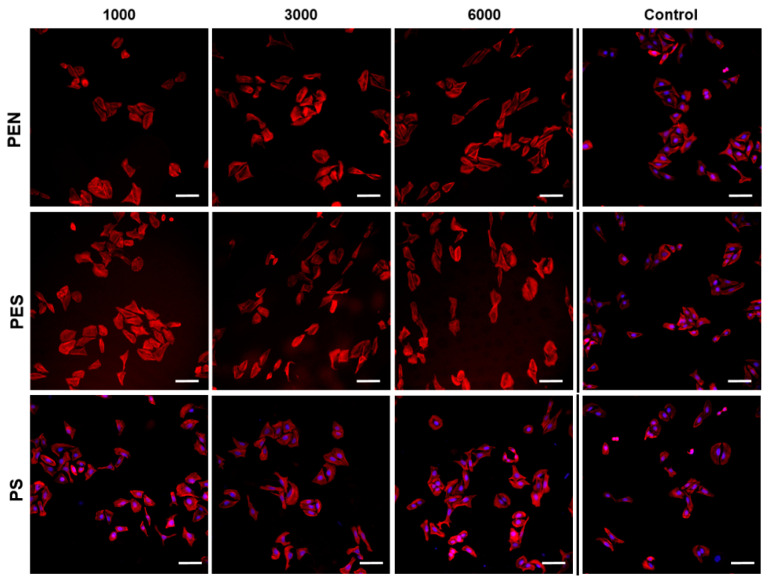
Fluorescence microscopy images of U-2 OS cells (human cells derived from osteosarcoma) growing on laser-treated PEN, PES, and PS with the fluence of 10 mJ·cm^−2^ and 1000, 3000, and 6000 pulses. The photos were acquired 24 h post-seeding. As a control, tissue culture polystyrene (TCPS) was used. The white line represents 20 μm.

The study of cell interaction with LIPSS patterns was the main idea of this study. Human U-2 OS cells were chosen as a model cell line for cytocompatibility measurement of the prepared materials. The fluorescence microscopy images for the cells after 24 h from seeding for PEN, PES, and PS (see Figure 8) showed that the laser treatment had an impact on cell alignment. 

**Figure 9:**
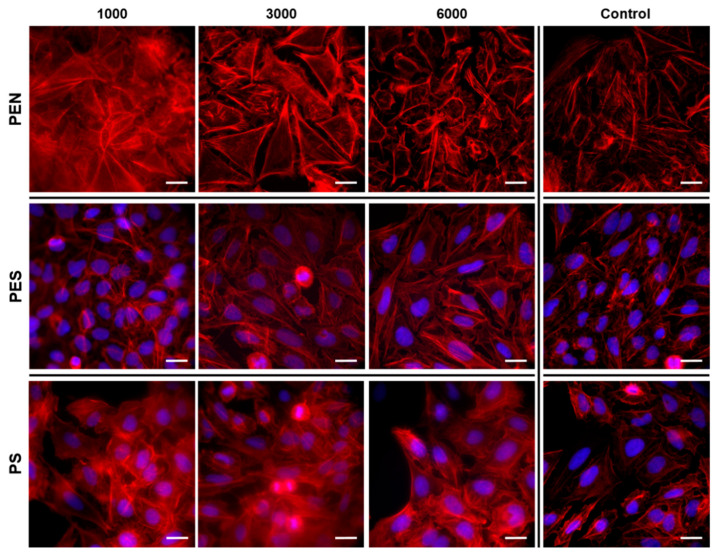
Fluorescence microscopy images of U-2 OS cells (human cells derived from osteosarcoma) growing on laser-treated PEN, PES, and PS with the fluence of 10 mJ·cm^−2^ and 1000, 3000, and 6000 pulses. The photos were acquired 72 h post-seeding. As a control, tissue culture polystyrene (TCPS), was used. The white line represents 20 μm.

**Figure 10:**
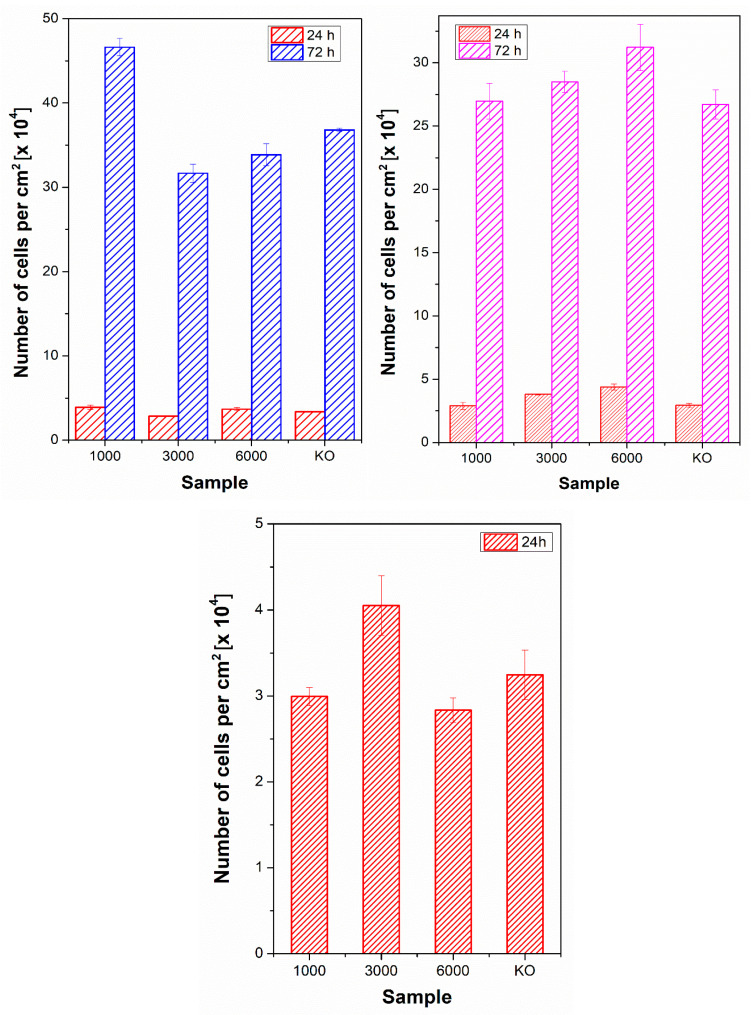
The number of adhered (24 h) and proliferated (72 h) U-2 OS cells on laser-treated polyethersulfone (PES, left image) and polystyrene (PS, right image) with the fluence of 10 mJ·cm^−2^ and 1000, 3000, and 6000 pulses. Due to strong fluorescence, only results after 24 h are introduced for PEN substrate. Tissue culture polystyrene was used as a control (KO).

With an increasing number of laser pulses, the cell shape was significantly affected, especially on PEN samples treated with 6000 pulses. The change in cell shape on polymers after application of 6000 pulses was observed for all studied samples, and there was a significant extension of the cells in one direction, corresponding with the LIPSS pattern formed. The changes in cell shape were preserved also after 72 h from cell seeding, as is obvious in detailed images for U-2 OS cells on PES, PEN, and PS in Figure 9 (larger photos are introduced in the Supplementary Materials). It is also apparent from Figure 9 and Figure 10 that the laser treatment had a positive effect on cell proliferation. The images of particular PEN and PES samples revealed that a homogeneously covered surface with U-2 OS cells was constructed.

From the plot in Figure 10, it is evident that after 24 h post-seeding, there was no statistically significant difference in the number of cells adhered on modified or unmodified samples compared to the control sample of tissue culture polystyrene (TCPS). It can be caused by the cell adaptation process to the samples after their seeding. After additional cultivation, 72 h post-seeding, cell proliferation was similar or even better compared to TCPS. The optimal cell number (significantly higher compared to pristine) was observed for PES after short laser exposure with 1000 pulses and for PS after long laser exposure with 6000 pulses. For PEN samples, we present only the results from the adhesion stage (after 24 h) since the high cell number and very high autofluorescence of PEN in the DAPI region did not enable us to determine the cell number based on the number of cell nuclei.

**Figure 11:**
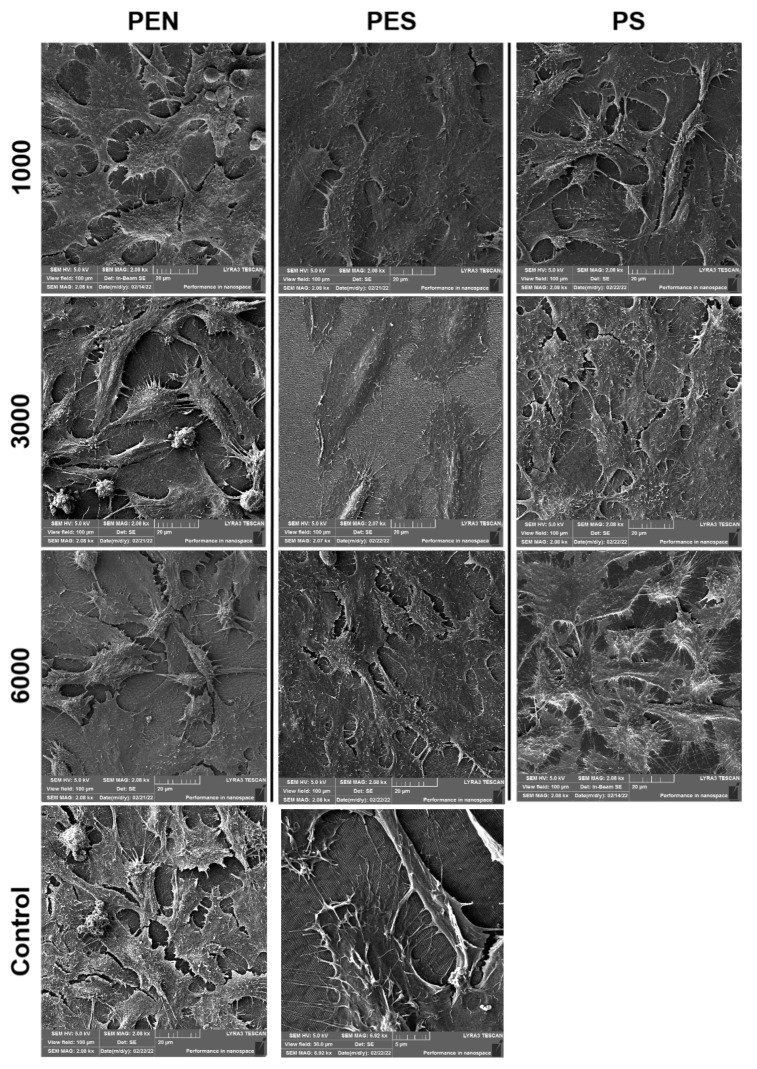
The SEM scans U-2 OS cells (72 h from seeding) on laser-treated PEN, PS, and PES with the fluence of 10 mJ·cm^−2^ and 1000, 3000, and 6000 pulses. Tissue culture polystyrene was used as a control (KO); the control is introduced at the bottom line with two different scanned areas, 100 × 100 and 30 × 30 μm^2^. A more detailed image for PES and 6000 pulses is introduced in the bottom right image.

We have also analyzed the SEM scans of U2-OS samples which were proliferated on the studied polymers treated with a different number of laser pulses (Figure 11). The SEM images clearly show the influence of the cell shape; the shape extension in the direction of the LIPSS pattern is most pronounced for polymers treated with 6000 pulses and PEN and PES polymers treated with 3000 pulses. 

## 3. Materials and Methods

### 3.1. Materials and Chemicals

The following polymer substrates with 50 μm thickness were used for excimer exposure and the following control of cell guidance: polyethylene naphthalate foil (PEN, T_m_ ~ 250–290 °C, T_g_ ~ 120 °C), polyethersulfone (PES, Goodfellow Cambridge Ltd., UK, the density of 1.37 g·cm^−3^), and polystyrene (PS, 1.05 g·cm^−3^, T_m_ ~ 240 °C, T_g_ ~ 100 °C, Goodfellow Cambridge Ltd., UK). 

### 3.2. Exposure of Substrates

The polymer samples were irradiated with a KrF laser (Coherent Inc., Santa Clara, CA, USA) Leap 100 K, the wavelength of 248 nm, pulse duration of 20–40 ns, repetition rate of 10 Hz) with a stabilized max. energy range of 900–1000 mJ and beam dimensions of 32 × 13 mm^2^ (maximum achievable values at the laser output). For the sake of illumination homogeneity and polarizer dimensions, only the central part of the beam profile, defined by an aperture of 10 mm × 10 mm, was used for the irradiation. The samples were mounted onto a translation stage and scanned at an angle of the beam incidence of 0° concerning the sample surface normal. Based on our previous experiments [17,28,29], we have chosen the laser fluence used in the present experiment as 10 mJ·cm^−2^, and the number of pulses was chosen in the range of 1000 to 6000 with the frequency of 10 Hz. The optical table Performance 07OTM501 (Melles Griot, Rochester, USA) with air pressure triggers was used. For the polarization, the cube of UV grade fused silica 25 mm × 25 mm × 25 mm with an active polarization layer was used. The stage with the samples was directed by Laser XY software, the motorized shift NRT100/M2 (Melles Griot).

### 3.3. Characterization of Substrates

The surface morphology, roughness, and area of the modified FEP were determined by atomic force microscopy (AFM) using Dimension ICON (Bruker Corp., Billerica, Massachusetts, USA). The samples were analyzed in Scan-Assyst mode using nitride lever SCANASYST-AIR with Si tip (spring constant of 0.4 N·m^−1^). NanoScope Analysis software was applied for data processing. The surface topography of the LIPSS shape and its dimensions were determined by the scanning electron microscope (SEM) using LYRA3 (Tescan, Brno, Czech Republic). The applied acceleration voltage for SEM was 10 kV. The substrates were covered with a Pt conductive layer with a thickness of 20 nm using a diode sputtering method (Quorum Q300T).

Detailed morphology of human U-2 OS (derived from osteosarcoma) cells adhered and growing on pristine and laser-modified polymers was examined by scanning electron microscopy (SEM) 72 h after seeding. The cells were washed three times with PBS (pH = 7.4), fixed by Karnovsky solution in cacodylate buffer, and dehydrated with ethanol (50, 70, 80, 90, and two times 99.9% for 10 min.). As a next step, hexamethyldisilazane (HDMS) was used to further dehydrate the samples (two times 10 min.). The cells intended for analysis by SEM were coated with a thin layer of Pt (20 nm) and analyzed by TESCAN LYRA3 GMU (Tescan, CZ) in a secondary-electron mode.

For elemental concentration, energy-dispersive X-ray spectroscopy (EDX) was used together with an F-MaxN analyzer and SDD detector (Oxford Instruments, Abingdon, UK). The applied acceleration voltage for EDX was 10 kV. The substrates were covered with a Pt conductive layer with a thickness of 20 nm using a diode sputtering method (Quorum Q300T).

The wettability of the polymer surface was studied goniometrically by measuring the contact angle. The contact angle was measured by the Surface Energy Evaluation System (SEE System, Brno, Advex Instruments, Czech Republic); drops of 8.0 ± 0.2 µL volume of distilled water were applied to the samples by an automatic pipette and the consequent photographs were evaluated (8 per sample).

X-ray photoelectron spectroscopy (XPS) was used for the analysis of the chemical composition of studied polymers. For analysis, an ESCAProbeP spectrometer (Omicron nanotechnology GmbH, Taunusstein, Germany) with a monochromatic X-ray beam source with an energy of 1486.7 eV was used. The samples were analyzed stepwise with a step of 0.05 eV perpendicularly concerning the orientation of the sample. The atomic concentration of elements (at.%) was evaluated by the CasaXPS software. 

### 3.4. Cell Cultivation 

Human cells derived from osteosarcoma (U-2 OS) from American Tissue Culture Collection (Manassas, VA, USA) were used to assess the cytocompatibility of PES, PEN, and PS matrices prepared in this study. The U-2 OS cells were cultivated in high-glucose Dulbecco’s modified Eagle medium (DMEM; Sigma-Aldrich, St. Louis, MO, USA) supplemented with a stable L-glutamine dipeptide and 10% fetal bovine serum (FBS; Thermo Fisher Scientific, Waltham, MA, USA). The U-2 OS cells were maintained in the exponential phase of growth at 37 °C, 5% CO_2_ atmosphere, and 95% humidity.

### 3.5. Sample Preparation for Fluorescence Microscopy

The material cytocompatibility was evaluated using U-2 OS cells from passages 5–9 (from cell defrosting after their delivery). First, the prepared samples were sterilized using 70% (*v*/*v*) ethanol in water for 30 min., after which they were inserted into cultivation plates with 12 wells (ø 2.14 cm; VWR, Radnor, PA, USA), washed with phosphate-buffered saline (PBS), and weighted by hollow cylinders from polymethyl methacrylate (Zenit, Prague, The Czech Republic). After that, 15,000 U-2 OS cells per 1 cm^2^ were seeded into each well in 1 mL of DMEM. Tissue culture polystyrene served as a control. The samples were prepared in triplicate. The cultivation endpoints were 24 and 72 h; then, the cultivation medium was removed, and the cells were washed with PBS and fixed with a 4% formaldehyde (methanol-free) solution in PBS for 20 min. at laboratory temperature in the dark. After that, the fixative was discarded and the cells were washed with PBS twice, which was followed by cell nuclei and F-actin staining using 0.5 µg·mL^−1^ of 4′,6-diamidino-2-phenylindole (DAPI; Sigma-Aldrich, St. Louis, MO, USA) and 2 µg·mL^−1^ of phalloidin conjugated to Atto 565 (Sigma-Aldrich, St. Louis, MO, USA), respectively, in PBS for 15 min. Then, the cells were washed twice with PBS and maintained in the dark until microscopy.

### 3.6. Fluorescence Microscopy

The fixed samples of U-2 OS cells were examined by wide-field fluorescence microscopy. For this, an inverse Olympus IX-81 microscope (Olympus, Tokyo, Japan) with xCellence software was used. The stained cells were captured by 10×, 20×, and 40× objectives with the NAs of 0.30, 0.45, and 0.60, respectively. F-actin and cell nuclei were monitored using a triple quad filter DAPI/FITC/TRITC (Olympus, Tokyo, Japan) and the regions of interest were captured by an EM-CCD camera (Hamamatsu, Honshu, Japan). The fluorescence images were deconvolved (50%), background-corrected, and the fluorescence channels (DAPI and TRITC) were merged, when possible, i.e., for PS samples and controls. Since PES and PEN exhibit high background autofluorescence in the DAPI channel, it was not possible to efficiently capture cell nuclei of cells growing on these two materials. Then, the cell number was counted using Image J software 1.53.

## 4. Conclusions

We were able to prepare LIPSS patterns on different aromatic polymers: PEN, PES, and polystyrene. The number of laser pulses for particular laser fluences was confirmed to play a crucial role in the pattern formation; e.g., the surface wettability and chemistry have altered the process of LIPSS formation. The material surface morphology and parameters such as effective surface area, pattern width, and height were confirmed both by atomic force microscopy and scanning electron microscopy. A dramatic increase in surface oxygen was detected for polystyrene treated with an excimer laser by the XPS technique. The increase in oxygen concentration was also observed for PEN polymer, and the main increase was observed for 1000 pulses; with further increases in several laser beam pulses, only a mild increase in oxygen concentration on the PEN surface was observed. On the contrary, for PES polymer, even an increase in oxygen after exposure with 1000 pulses was observed. The LIPSS pattern was confirmed to be a significant tool for the cell alignment process. The laser-treated substrates witl LIPSS have shown support for both cell adhesion and proliferation of human osteoblasts (U-2 OS) and ability to guide the cell growth.

## Figures and Tables

**Table 1:** Oxygen atomic concentrations for pristine and laser-treated polystyrene, polyethylene naphthalate, and polyethersulfone acquired from XPS spectra. The laser exposure of 10 mJ·cm^−2^ with 1000, 3000, and 6000 pulses was applied.

	Pristine	1000	3000	6000
PS	0	3.5	15.6	25.2
PES	20	22.9	19.8	20.8
PEN	19.8	27.3	31.8	31.9

## Data Availability

Data are contained within the article.

## Supplementary Materials

The following supporting information can be downloaded at: https://www.mdpi.com/article/10.3390/ijms23094676/s1.
