# Supplementary material for: Mammalian Cell Interaction with Periodic Surface Nanostructures
Source: Int J Mol Sci. 2022 Apr 23;23(9):4676. doi: 10.3390/ijms23094676 (PMC9100987; doi:10.3390/ijms23094676)
Supplement: Supplementary file 1 [file ijms-23-04676-s001.zip › ijms-1650523-supplementary.pdf]

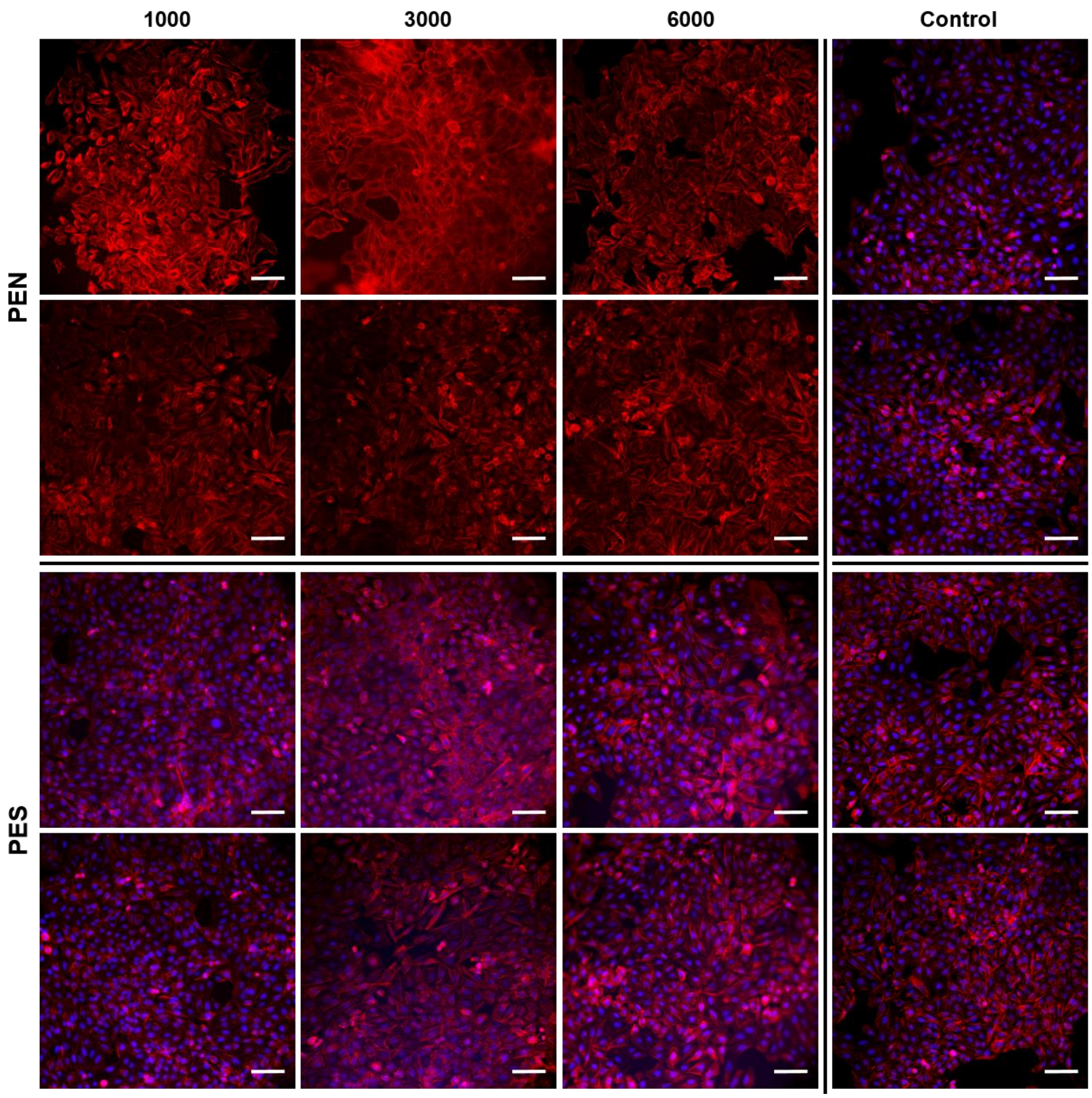

Figure S1. Fluorescence microscopy images of U-2 OS cells (human cells derived from osteosarcoma) growing on laser-treated PEN and PES with the fluence of 10 mJ·cm^{-2} and 1,000, 3,000, and 6,000 pulses. The photos were acquired 72 h post-seeding. As a control, tissue culture polystyrene (TCPS), was used. The white line represents 80 μm.
